# Special Issue “Pathophysiology and Treatment of Alzheimer’s Disease”

**DOI:** 10.3390/ijms25116015

**Published:** 2024-05-30

**Authors:** Jeffrey Fessel

**Affiliations:** Department of Medicine, University of California, San Francisco, 2069 Filbert Street, San Francisco, CA 94123, USA; jeffreyfessel@gmail.com; Tel.: +1-415-563-0818

## 1. Introduction

The majority of clinical trials, whose primary aims were to moderate Alzheimer’s dementia (AD), have been based upon the prevailing paradigm, i.e., the amyloid hypothesis. None of those trials have cured AD, whereas the goal of treatment for AD is, or should be, the reversal of dementia, i.e., its cure. The multiple failures of all of those trials make a strong suggestion that the prevailing paradigm is incorrect either in whole or in part. If incorrect in part, the implication is that therapy addressing amyloid may require supplementation with therapy aimed at addressing additional causal factors [1]. There is, however, another approach for curing AD that is described here.

This article shows 21 causal elements that contribute to the pathogenesis of AD. Lithium addresses 16 of the 21; four of the remaining five are addressed by fluoxetine, leaving one unaddressed. Adding a weekly injection of dulaglutide will be beneficial and may even cure AD, because this drug is an agonist of GLP-1, which is present in all brain cells [2]. The elements not addressed by lithium and fluoxetine are hypertension and nutrient deficiencies, primarily folate and vitamin D. If hypertension is present in individual patients, it requires long-term treatment with antihypertensive drugs; if deficiencies of folate and vitamin D are present, these require short-term correction.

For therapy to be effective, its duration must be estimated, although a definitive time can be established only through clinical trials: until this is accomplished, six months seems a reasonable duration. A clinical trial to test the efficacy of a potentially curative treatment must include an equipoise arm, plus a predetermined amount above that attained by the equipoise arm, which might lead to the declaration of a successful curative treatment. In order to match the experimental therapy, the equipoise arm should contain three drugs, e.g., lecanemab, memantine, and pioglitazone. Finally, if within three months either before or after the diagnosis of dementia, known risk factors for dementia either newly appear or, if previously present, become distinctly worse, then treating them before providing experimental therapy might reverse the dementia.

Table 1 shows 21 elements that are known to contribute to the pathogenesis of AD; lithium addresses 16 of the 21 [3,4,5,6,7,8,9,10,11,12,13,14,15,16,17,18,19]. The five exceptions are hyperlipidemia, hypertension, TGF-ß, nutrient deficiencies, and metabolic syndrome, and, although most reports show that lithium ameliorates the deposition of Aß [14,15,20,21], one report found otherwise [22]. However, fluoxetine benefits TGF-β deficiency [23,24], hyperlipidemia [25], and metabolic syndrome [25]; additionally, it prevents the aggregation of Aβ [26]. Thus, most cases of AD should be cured after six months of taking the combination of lithium, at a dose of 150 mg qd, fluoxetine, at a dose of 20 mg qd, and one weekly subcutaneous injection of dulaglutide, at a dose of 1.5 mg.

The efficacy of lithium in addressing the causal elements in Table 1 is unexpectedly broad. Therefore, the following is a very brief description of lithium’s efficacy in addressing each element in Table 1, as reported in each cited article. It is followed by descriptions of the benefits of fluoxetine and dulaglutide.

Lithium treatment abolished the GSK-mediated increase in Aβ and reduced the plaque burden in the brains of AD model mice [20]; also in AD model mice, it decreased the ɤ cleavage of the amyloid precursor protein and, therefore, the production of Aβ and amyloid plaques [21]. Others found that lithium did not significantly decrease the Aβ load but did decrease the phosphorylation of tau protein [22]. Lithium enhanced autophagy in cells over-expressing TGF-β [4], and did so by decreasing inositol-1,4,5-triphosphate (IP_3_) levels via the inhibition of inositol monophosphatase [3]. In pluripotent stem cells homozygous for ApoE4, which were pre-treated with lithium and then challenged with glutamate, there was significantly less elevation of intracellular Ca^2+^ compared to cells heterozygous for ApoE3 [5]. Regarding circadian rhythm, rhythmic clock-controlled genes were more responsive to lithium than non-rhythmic genes [6], and a systematic review showed an association between the use of lithium and ‘morningness’, which is a chronotype indicating a preference for more alertness and activity in the morning, contrasted with ‘eveningness’, which is a preference for more alertness and activity in the evening [7]. In the treatment of both acute and chronic depression, lithium was more effective than a placebo as an adjunct to antidepressants [8]. In diabetic patients treated with oral hypoglycemic agents or insulin, fasting and one hour post-prandial blood glucose levels were both significantly decreased by lithium [9]. At microRNA (miRNA) loci, lithium induced epigenetic modifications affecting DNA methylation, histone acetylation, and common variants [10]. Regarding inflammation, three months of treatment with lithium caused a decreased number of cells secreting IL-6 and IFNɤx [11]. Lithium restored sensitivity to insulin by acting on several stages of the PI_3_K/Akt insulin-signaling pathway [12]. Clinically relevant concentrations of lithium raised the mitochondrial levels of the respiratory chain complexes 1 + 111 and 11 + 111 [13]; it increased intracellular protein expression by 10% and 14% in cortical neurons and hippocampal neurons, respectively, and extracellular BDNF increased by 428% in cortical neurons and by 44% in hippocampal neurons [14]. A high-dose lithium treatment increased the generation of neuroblasts and neurons in human hippocampal progenitors [15]. In hippocampal slices, lithium increased the amplitude of the presynaptic fiber volley and enhanced excitatory synaptic transmission in CA1 pyramidal cells [16]. Lithium also expanded the pool of hippocampal progenitor cells and promoted their differentiation into neurons [19]. Lithium led to weight gain in a high proportion of patients treated, with up to a quarter becoming clinically obese; the mechanism for this weight gain is unknown [31]. Among 1885 subjects with an initial diagnosis of bipolar disorder, 4.6% had had a stroke; this affected only 2.8% of those using lithium but 5.4% of those not using lithium, giving a hazard ratio (HR) of 0.39 for the use of lithium. For those with the highest and longest cumulative exposures to lithium, the HR equaled 0.25 and 0.20, respectively [17]. Wnt signaling was inhibited by glycogen synthase kinase-3β (GSK-3β) and lithium prevented that inhibition [18], which, in turn, prevented the inhibition of β-catenin and promoted lithium’s proliferation of hippocampal progenitor cells [19].

Fluoxetine has efficacy for items in Table 1 that are either not fully or only partially covered by lithium. In a human study, fluoxetine administered for 16 weeks produced reductions of 9.6% in both total cholesterol and low density lipoprotein (LDL) cholesterol [25]. Other relevant studies were only possible in mice or in cell cultures. Fluoxetine administration, started seven days before mice were injected with Aβ into a cerebral ventricle, prevented memory loss, as well as both the loss of hippocampal TGF-β and decrements of synaptophysin and PSD95 [23]. A study that measured the aggregation of Aβ_42_ showed that fluoxetine binds to Aβ and prevents its aggregation into amyloid [26]. Fluoxetine administered to a mouse model of AD enhanced the levels of the protein phosphatase 2A, which, in turn, raised the level of active β-catenin, causing a beneficial result; it also lowered levels of Aβ by reducing the cleavage of APP, preventing neuronal apoptosis [32]. The neuroprotection from fluoxetine is partly caused by the release of TGF-β from astrocytes. At therapeutic concentrations (100 nM–1 μM), fluoxetine significantly prevented Aβ-induced toxicity in mixed glia-neuronal cultures, but not in pure neuronal cultures, whereas the medium collected from cultured astrocytes that had been challenged with fluoxetine protected pure cortical neurons against Aβ toxicity [24].

Regarding dulaglutide, it is important to note that all brain cells have GLP-1 ligands [33,34,35,36,37,38,39], so their functions may be addressed by agonists of GLP-1. Furthermore, because drugs exert their effects in the brain parenchyma, it is also important to differentiate between their presence in the parenchyma and their presence within brain capillaries. For the commercially available GLP-1 agonists, the percentage in the brain parenchyma versus in the brain capillaries (in which drugs are ineffectual) was highest, at 61.8%, for dulaglutide; the relative brain uptake, compared to dulaglutide, was only 28% for exenatide, 14% for lixisenatide, and virtually zero for liraglutide, semaglutide, and tirzepatide [40]. Those percentages derive from the relative rates (Ki) of significant brain uptake one hour after IV injection. There is only one study of dulaglutide and cognition in humans, but it was a very large study and produced convincing results, possibly due to dulaglutide’s excellent uptake of 61.8% in the brain parenchyma. This very large study of dulaglutide was a randomized, double-blind placebo-controlled trial of subjects aged ≥50 years, with either established or newly diagnosed type 2 diabetes and additional cardiovascular risk factors; cognitive function was assessed at baseline and during follow-up using the Montreal Cognitive Assessment (MoCA) and digit symbol substitution test (DSST) [41]. During a median follow-up of 5.4 years, 8828 participants provided a baseline and one or more follow-up MoCA or DSST scores, of whom 4456 had been assigned dulaglutide and 4372 had been assigned a placebo. After adjusting for individual standardized baseline scores, the hazard of substantive cognitive impairment was reduced by 14% in those assigned dulaglutide (HR 0·86, *p*= 0·0018). The mechanism behind this benefit may be that dulaglutide reduced the disadvantageous hyperphosphorylation of tau and neurofibrillary tangles, via improving the PI3K/AKT/GSK3β signaling pathway [42].

## 2. Discussion

In light of the data described in this editorial, it is probably an approximately correct conclusion that most cases of AD might be cured within six months of taking triple therapy, consisting of lithium, at a dose of 150 mg daily, fluoxetine, at a dose of 20 mg daily, and one weekly injection of dulaglutide at a dose of 1.5 mg, which, between them, address 19/21 of the causal factors in Table 1. The causal elements not addressed by the suggested combination are hypertension and inadequate levels of folate and vitamin D; regardless, if these are present in individual patients, they should be treated with appropriate antihypertensives and with folate and vitamin D.

A clinical trial is emphatically required to establish the validity, clinical relevance, and safety of the proposed triple drug treatment. The equipoise treatment with three drugs could be lecanemab, memantine, and pioglitazone (which prevents the phosphorylation of JAK–STAT in astrocytes and thereby induces increases in neurons, oligodendrocytes, and endothelial cells, and decreases in microglia [43,44].

## Figures and Tables

**Table 1:** Elements that participate in the pathogenesis of Alzheimer’s dementia.

Elements	Refs.
Aβ deposition ↑	[20,21]
Tau deposition ↑	[22]
ApoE4ɛ ↑	[5]
Autophagy ↓	[3,4]
Ca^2+^ dysregulation ↑	[5]
Circadian dysrhythmia ↑	[6,7]
Depression ↑	[8]
Diabetes ↑	[9]
Epigenetics ↑	[10]
Inflammation ↑	[11]
Hypertension	
Hyperlipidemia	[25]
Insulin resistance ↑	[12]
Metabolic syndrome ↑	
Mitochondrial dysfunction ↑	[27]
Neuronal/synaptic dysfunctions ↑	[14,15,16]
Nutrient (folate and vitamin D deficiencies) ↑	
TGF-β deficiency ↑	
Underweight ↑	[28,29,30]
Vascular pathologies ↑	[17]
Wnt/catenin-β ↓	[18,19]

↑ or ↓ indicate direction of change before treatment. Refs indicate citations for the treatment.
